# Characterization of a model of systemic inflammation in humans *in vivo* elicited by continuous infusion of endotoxin

**DOI:** 10.1038/srep40149

**Published:** 2017-01-05

**Authors:** D. Kiers, R. M. Koch, L. Hamers, J. Gerretsen, E. J. M. Thijs, L. van Ede, N. P. Riksen, M. Kox, P. Pickkers

**Affiliations:** 1 Radboud university medical center, 6500 HB, Radboud Institute for Molecular Life Sciences, Department of Intensive Care Medicine, 6500 HB, Nijmegen, The Netherlands; 2 Radboud university medical center, 6500 HB, Radboud Institute for Molecular Life Sciences, Department of Pediatrics, 6500 HB, Nijmegen, The Netherlands; 3 Radboud university medical center, 6500 HB, Radboud Institute for Molecular Life Sciences, Department of Internal Medicine, 6500 HB, Nijmegen, The Netherlands

## Abstract

Investigating the systemic inflammatory response in patients with critical illness such as sepsis, trauma and burns is complicated due to uncertainties about the onset, duration and severity of the insult. Therefore, *in vivo* models of inflammation are essential to study the pathophysiology and to evaluate immunomodulatory therapies. Intravenous bolus administration of endotoxin to healthy volunteers is a well-established model of a short-lived systemic inflammatory response, characterized by increased plasma cytokine levels, flu-like symptoms and fever. In contrast, patients suffering from systemic inflammation are often exposed to inflammatory stimuli for an extended period of time. Therefore, continuous infusion of endotoxin may better reflect the kinetics of the inflammatory response encountered in these patients. Herein, we characterize a novel model of systemic inflammation elicited by a bolus infusion of 1 ng/kg, followed by a 3hr continuous infusion of 1 ng/kg/h of endotoxin in healthy volunteers, and compared it with models of bolus administrations of 1 and 2 ng/kg of endotoxin. The novel model was well-tolerated and resulted in a more pronounced increase in plasma cytokine levels with different kinetics and more prolonged symptoms and fever compared with the bolus-only models. Therefore, the continuous endotoxin infusion model provides novel insights into kinetics of the inflammatory response during continuous inflammatory stimuli and accommodates a larger time window to evaluate immunomodulating therapies.

Systemic inflammation is a common hallmark of patients with sepsis^1^, trauma^2^, burns^3^, as well as those undergoing major surgery^4^. This inflammatory response follows the activation of specific receptors by pathogen-, or danger associated molecular patterns (PAMPs and DAMPs), and is characterized by the production of cytokines and chemokines. In turn, these cytokines and chemokines trigger complement activation and coagulation, leukocyte migration, and increased vascular permeability^5^. A dysregulated immune response can have detrimental effects, such as hemodynamic instability, organ dysfunction, and prolonged immune suppression^6^.

A comprehensive appreciation of the innate immune response is crucial to understand the pathogenesis of systemic inflammation observed in patients. However, investigating the innate immune response in patients is hampered by heterogeneity, uncertainties about the moment of onset and, in the case of sepsis, the accuracy of the diagnosis^6^. In order to overcome these impediments, models of systemic inflammation may facilitate investigations into the pathophysiological mechanisms and evaluate the effects of immunomodulatory interventions. As *in vitro* and animal models have obvious drawbacks concerning extrapolation to humans^7^, investigating systemic inflammation and immunomodulation is of great importance^8^.

A controlled, transient and reproducible systemic inflammatory response in humans can be achieved with an intravenous (i.v.) bolus administration of endotoxin (lipopolysaccharide (LPS)), a compound derived from the cell membrane of Gram-negative bacteria. This inflammation is characterized by flu-like symptoms, fever, hemodynamic alterations and increased plasma levels of cytokines^9^. Previous studies have shown a dose-dependent effect of endotoxin on the magnitude of the inflammatory response, but largely unaltered kinetics with increasing endotoxin dosages^10^,^11^,^12^. Furthermore, repeated endotoxin administration studies have demonstrated the development of endotoxin tolerance, representing an immune suppressed state that resembles sepsis-induced immunoparalysis^13^. This experimental human endotoxemia model has been frequently used to investigate the pathophysiology of systemic inflammation, to assess a broad range of immunosuppressive and immunostimulatory drugs and non-pharmacological interventions^14^,^15^,^16^,^17^, and to evaluate the effects of inflammation on other biologic processes, such as iron homeostasis^18^.

Patients suffering from systemic inflammation are in most cases exposed to inflammatory stimuli for extended periods of time^19^. Therefore, a bolus administration of endotoxin likely poorly reflects the kinetics of the inflammatory response observed in these patients^9^,^19^, and a continuous infusion of endotoxin may provide a more accurate representation. Furthermore, as the endotoxin-induced inflammatory response is rapidly orchestrated and waned after bolus administration, investigational treatments are typically initiated before endotoxin administration. Although this approach increases the likelihood to show efficacy of the intervention in the experimental model, it does not reflect the clinical situation in which treatment is initiated after systemic inflammation has presented. An inflammatory model that develops over several hours may provide a larger time window to evaluate interventions once inflammation has developed.

Taken together, the pathophysiology of systemic inflammation and immunomodulatory treatments are difficult to study in patients. The human endotoxemia model that is currently used is characterized by a rapid and short-lived systemic inflammatory response after a bolus administration of endotoxin. A continuous infusion of endotoxin may provide more insight into the kinetics of the inflammatory response in patients with ongoing inflammation.

Herein, we characterize a novel model of systemic inflammation elicited by continuous endotoxin infusion (an initial endotoxin bolus of 1 ng/kg followed by 1 ng/kg/h for 3 hours). Additionally, we provide a context to interpret the characteristics of the continuous infusion model by describing the characteristics of endotoxin models using 1 or 2 ng/kg bolus administrations and responses to placebo administration. We report the kinetics of plasma cytokines, hemodynamic alterations, symptoms and leukocyte changes, and explore the coherence between different cytokines and their association with clinical parameters.

## Results

### Demographic characteristics, elicitation of systemic inflammation and safety

Eligible subjects were healthy, non-smoking male subjects, aged 18–35 years. Demographic characteristics of the subjects are listed in Table 1. All subjects were Caucasian. There were no differences in baseline characteristics between the groups. All subjects received a venous and arterial cannula to accommodate infusion of fluids and endotoxin and frequent blood withdrawal and continuous blood pressure monitoring. Systemic inflammation was elicited by either continuous endotoxin infusion (an initial endotoxin bolus of 1 ng/kg, followed by 1 ng/kg/h for 3 hours) or a bolus administration of 1 or 2 ng/kg bolus administrations of endotoxin. The placebo group received a comparable volume of NaCl 0.9%. No serious adverse events occurred and all subjects were well at the time of discharge, eight hours after initiation of endotoxin administration.

### Plasma cytokines and chemokines

**Figure 1:**
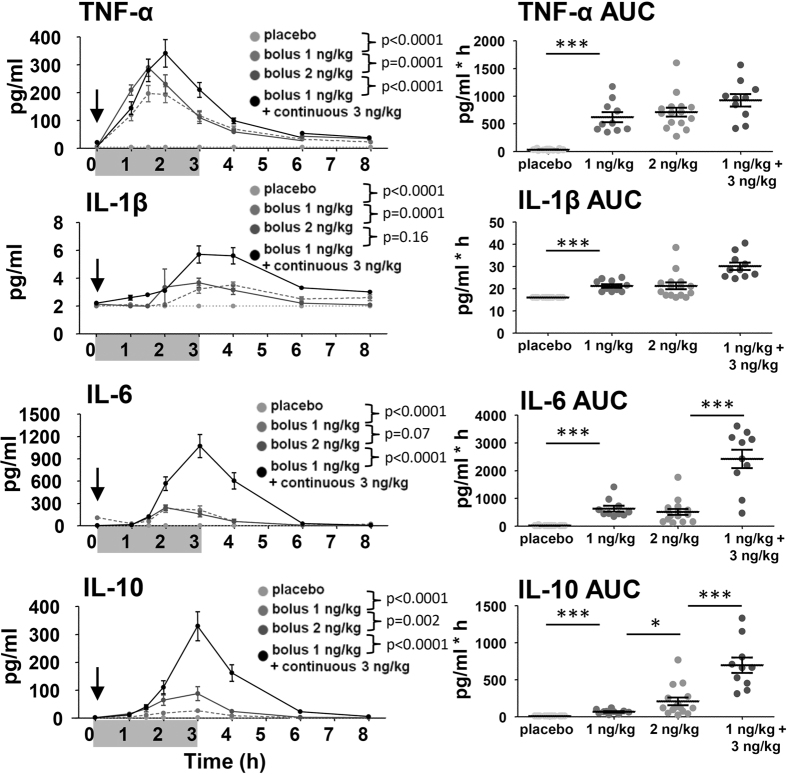
Kinetics and total production of cytokines. In the left panels, plasma concentrations of Tumor Necrosis Factor (TNF)-α, Interleukin(IL)-1β, IL-6 and IL-10 over time are depicted. The arrow represents the time of bolus endotoxin administration, the grey bar represents the period of endotoxin infusion in the continuous endotoxin group. Data are expressed as mean ± SEM pg/mL. Differences between groups were evaluated using 2-way ANOVAs (of time × group), and interaction term p-values are displayed. In the right panels, dot plots of the area under the time-concentration curve (AUC) with mean ± SEM are shown. Differences between groups were evaluated using Students t-tests. *p < 0.05, **p < 0.01, *p < 0.0001, ^#^p = 0.05–0.10.

**Figure 2:**
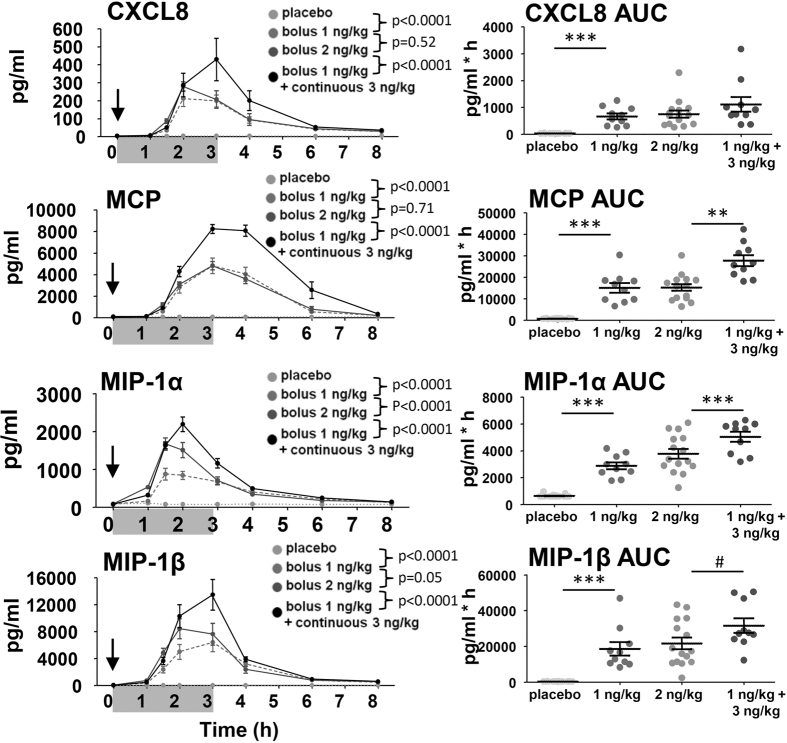
Kinetics and total production of chemokines. In the left panels, plasma concentration of CXC motif ligand (CXCL) 8, Monocyte Chemoattractant Protein (MCP) and Macrophage Inflammatory Protein (MIP)-1α and 1β over time are depicted. The arrow represents the time of bolus endotoxin administration, the grey bar represents the period of endotoxin infusion in the continuous endotoxin group. Data are expressed as mean ± SEM pg/mL. Differences between groups were evaluated using 2-way ANOVAs (of time × group), and interaction term p-values are displayed. In the right panels, dot plots of the area under the time-concentration curve (AUC) with mean ± SEM are shown. Differences between groups were evaluated using with Students t-tests. *p < 0.05, **p < 0.01, *p < 0.0001.

To determine serial plasma levels of cytokines Tumor Necrosis Factor (TNF)-α, interleukin (IL)-1β, IL-6 and IL-10 (Fig. 1) and chemokines (C-X-C-motif) ligand (CXCL)8 (also known as IL-8), Monocyte Chemoattractant Protein (MCP)-1, Macrophage Inflammatory Protein (MIP)-1α and MIP-1β, blood was frequently sampled throughout the experiment. All plasma cytokine concentrations were below detection limits in all subjects prior to endotoxin administration and significantly increased afterwards (Fig. 2). In the placebo group, no increases in any of the cytokines and chemokines were observed.

#### Kinetics of plasma cytokines and chemokines

In the continuous infusion group, the kinetics of all cytokines and chemokines were markedly different compared with bolus administration of 2 ng/kg (Figs 1 and 2). Of note, in the continuous infusion group plasma levels of TNF-α and MIP-1α already decreased before endotoxin infusion was stopped. The differences between 1 and 2 ng/kg bolus administration groups were less pronounced, and only reached statistical significance for TNF-α, IL-1β, IL-10 and MIP-1α.

#### Total cytokine and chemokines production

The total cytokine response, expressed as the area under the time-concentration curve (AUC), was higher for IL-1β, IL-6, IL-10, MCP-1 and MIP-1α in the continuous infusion group compared with the 2 ng/kg bolus group, but similar for TNF-α and CXCL8. There was a trend towards higher total production of MIP-1β in the continuous infusion group. Except for higher production of IL-10 in the 2 ng/kg bolus administration group, there were no differences in total cytokine production between the 1 and 2 ng/kg bolus groups.

### Clinical parameters

**Figure 3:**
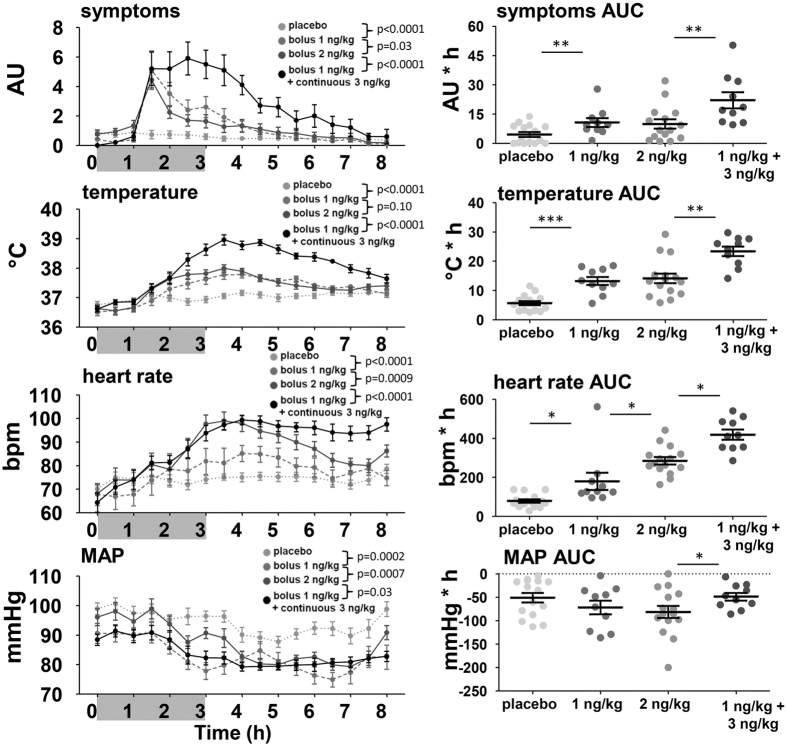
Kinetics and area under the curve of clinical parameters. In the left panel, change over time of symptoms (arbitrary units), temperature (°C), heart rate (beats per minute (bpm)), and mean arterial pressure (MAP) (mmHg) over time are depicted. The arrow represents the time of bolus endotoxin administration, the grey bar represents the period of endotoxin infusion in the continuous endotoxin group. Data are expressed as mean ± SEM. Differences between groups were evaluated using 2-way ANOVAs (of time × group), and interaction term p-values are displayed. In the right panel, dot plots of the area under the time-concentration curve (AUC) with mean ± SEM are shown. The AUC of temperature, heart rate and MAP was corrected for baseline. Differences between groups were evaluated using with Students t-tests. *p < 0.05, **p < 0.01, *p < 0.0001.

Endotoxin-induced flu-like symptoms (headache, nausea, shivering, muscle and back pain) reported by the subjects were scored every 30 minutes at 0–5 points. These symptoms remained at baseline in placebo-treated subjects, whereas the onset of flu-like symptoms was observed one hour after initiating endotoxin administration (Fig. 3). Continuous endotoxin infusion resulted in prolonged, but equally severe, flu-like symptoms in comparison to 2 ng/kg bolus administration. Small differences in the kinetics of the symptom scores were identified between the 1 and 2 ng/kg bolus administration groups. However, the total burden of symptoms, represented by the area under the time-symptom curve, was identical in these groups. The rise in temperature was markedly more pronounced in the continuous administration group compared with the 2 ng/kg bolus group, whereas there were no differences in the temperature response between the 1 and 2 ng/kg bolus administration groups (Fig. 3). Although an endotoxin-dose-dependent increase in heart rate was observed, mean arterial pressure decreased to a similar extent in all subjects, also in those who received placebo (Fig. 3).

### Hematological parameters

**Figure 4:**
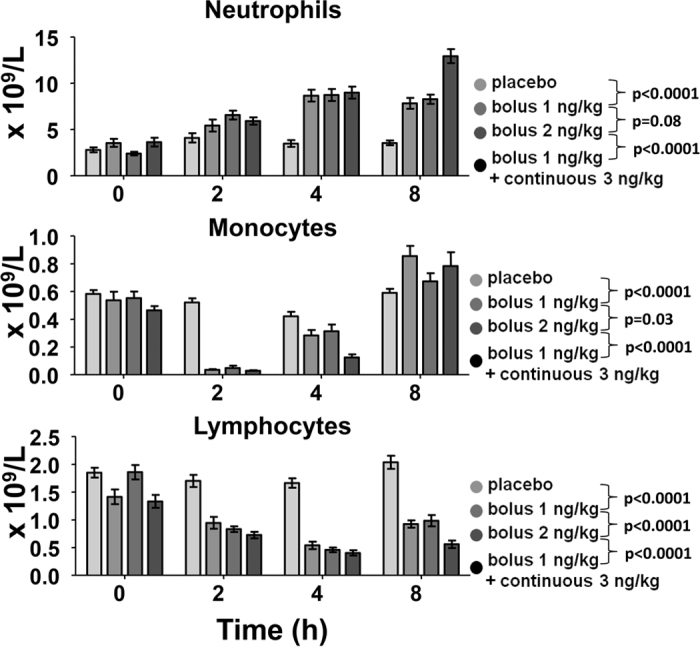
Time course of leukocytes. Bars represent mean ± SEM of circulating numbers of neutrophils, monocytes and lymphocytes. Differences between groups were evaluated using 2-way ANOVAs (time × group), and interaction term p-values are displayed.

Endotoxemia resulted in neutrophilia, and a mono- and lymphocytopenia, with the nadir at two and four hours, respectively (Fig. 4). These hematologic changes were more pronounced in the continuous infusion group compared with bolus administration of 2 ng/kg endotoxin. Neutrophilia was similar after a endotoxin bolus of 1 and 2 ng/kg, while the kinetics of circulating monocyte and lymphocyte numbers differed slightly, yet significantly.

### Hierarchical clustering and exploratory factor analysis

**Figure 5:**
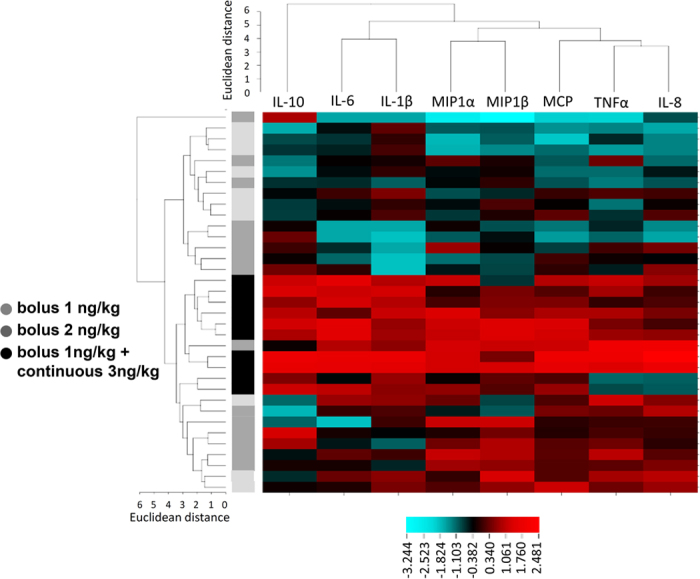
Hierarchial clustering of cytokines and chemokines and subjects. Each subjects occupies a row and each cytokine or chemokines occupies a column. The color code shows the normalized, log-transformed plasma concentration below (blue) or above (red) the mean, or at the median (black). The vertical axis on the left shows the color code of each subject classified as 1 ng/kg (light grey), 2 ng/kg (dark grey) or continuous infusion (black).

**Figure 6:**
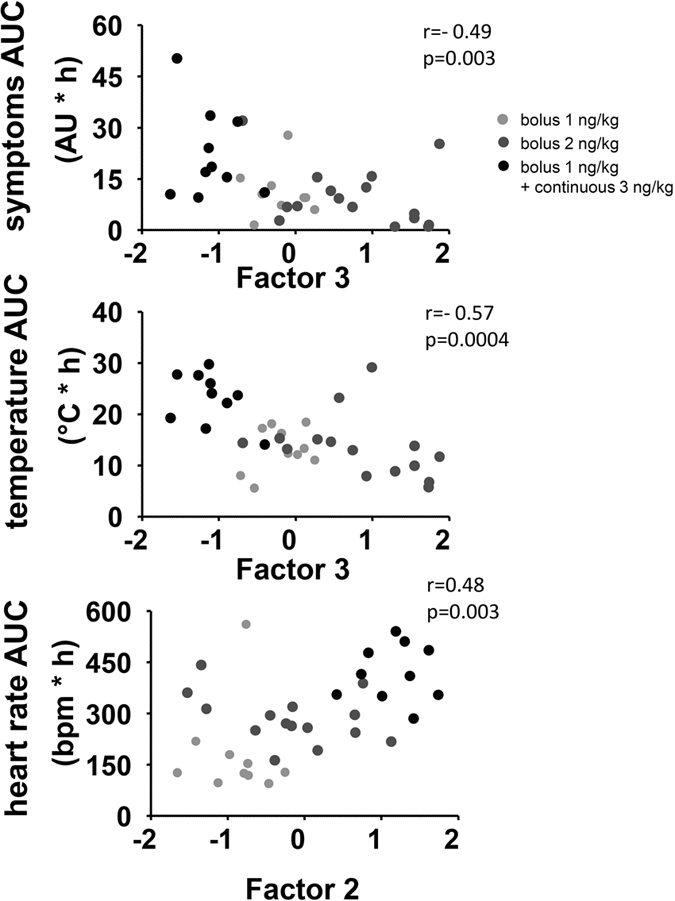
Correlations of factors with clinical parameters. Correlations between individual score on Factor 3 and the area under the curve (AUC), Factor 3 and symptoms (Panel A) and the baseline-corrected AUC of temperature (Panel B) and Factor 2 and the baseline-corrected AUC of heart rate (Panel C). R and p-values were calculated using Pearson correlations.

To identify relationships and similarities between cytokines and subjects, we performed hierarchical clustering analysis on cytokine responses and subjects. This analysis revealed a separate cluster for the anti-inflammatory cytokine IL-10, while three clusters were identified consisting of CXCL8, TNF-α and MCP-1, MIP-1α and MIP-1β, and IL-6 and IL-1β (Fig. 5). Subjects clustered into three distinct groups; those who received continuous infusion (middle rows), those with low cytokine responses (upper rows) after bolus administration, and those with high cytokine responses (lower rows) after bolus administration. To explore to what extent cytokines responses are related, we performed exploratory factor analyses. This is an unsupervised method that can identify latent constructs (factors) in a multi-dimensional data set. The loading of a variable on a factor represents the relative weight of the variable in the factor. Exploratory factor analyses revealed similar grouping of cytokines (Table 2); with high loadings of CXCL8 and TNF-α in factor 1, a high loading of IL-10 in factor 2, a high negative loading of IL-1β and IL-6 in factor 3, and high loadings of MIP-1α and MIP-1β in factor 4. These factors explain 92% of the variance in the dataset. Factor 3, which has high negative loadings of IL-1β and IL-6, correlated with symptoms and change in temperature (Fig. 6). As such, high values of IL-1β and IL-6 are associated with higher symptom severity and a larger increase in temperature. Change in heart rate correlated with factor 2, which has a high loading of IL-10.

## Discussion

Herein, we characterize a novel human *in vivo* model of systemic inflammation elicited by continuous endotoxin infusion. Furthermore, we provide a framework for interpretation of this model by also describing two models of systemic inflammation elicited by bolus administration of endotoxin. This novel model of continuous infusion resulted in a higher production of IL-6, IL-10, MCP-1 and MIP-1α, and different kinetics of all cytokines as well as circulating leukocyte numbers. Furthermore, it resulted in a more pronounced rise in temperature and heart rate, and prolonged duration of flu-like symptoms. As such, this novel model induces a more pronounced and sustained systemic inflammatory response. There were no relevant differences in cytokine responses, clinical parameters, and leukocyte kinetics between bolus administrations of 1 or 2 ng/kg endotoxin. In addition, unsupervised clustering and exploratory factor analysis revealed identical clusters of cytokines, some of which are clearly associated with clinical symptoms and fever.

We are the first to describe a model of continuous infusion of endotoxin at a relatively high dose of 1 ng/kg/h. Low dose endotoxin infusion of another *E coli* derived batch (G2 B274, 0.075 ng/kg/h) has been described previously^20^. At this lower dose, endotoxin infusion increased body temperature with 0.5 ᵒC, and cytokine levels peaked to 5–10 and 20–70 pg/mL for TNF-α and IL-6, respectively^20^. These changes are far more discrete compared with the higher dose continuous infusion model reported here, in which body temperature rises with 2.5 ᵒC and peak levels of TNFα and IL-6 are 340 pg/ml and 1000 pg/mL respectively. As such, low dose infusion mimics low-grade inflammation, as observed in chronic illnesses such as metabolic syndrome, atherosclerosis and type 2 diabetes^20^, whereas high dose infusion results in more profound systemic inflammation as observed in patients with sepsis. The cumulative dose of 4 ng/kg of endotoxin has been administered safely as a bolus in previous studies^10^,^11^,^12^. Therefore, no safety issues were expected, and indeed no serious adverse events occurred.

Previous studies have evaluated the dose-effects of different batches of endotoxin, in bolus administrations ranging from 0.5 ng/kg to 4 ng/kg^10^,^11^,^12^. These studies have shown that the extent of inflammation elicited by endotoxin administration is dose-dependent, but the difference between successive dosages may be too small to be significant. Similarly, we did not observe a clear dose-dependent effect concerning the 1 and 2 ng/kg endotoxin bolus administrations, although this was not the primary goal of this study. For most cytokines, the total response in the continuous model was higher compared to the 2 ng/kg bolus model. Although our study design does not allow to differentiate whether this is due to dose or infusion mode, these data indicate a more profound systemic inflammatory response using the continuous infusion protocol. However, in this context it is noteworthy that the total production of TNF-α and CXCL8 remained similar to that observed after bolus administration of 2 ng/kg endotoxin. TNF-α is known as the primary mediator in the inflammatory response and CXCL8 attracts and activates leukocytes to the site of infection^21^. Speculatively, both TNF-α and CXCL8 are under the control of a negative feedback mechanism that prevents unrestrained triggering of the inflammatory cascade, thereby prohibiting an uncontrolled pro-inflammatory response. This theory is supported by the observation that in the continuous endotoxin infusion group, plasma levels of TNF-α are already declining while endotoxin infusion continues. This phenomenon is also observed in patients with sepsis, in whom plasma levels of TNF-α decline rapidly and therefore do not represent disease severity^22^. In contrast to the restrained production of TNF-α and CXCL8, we observed a fourfold increase in IL-10 and IL-6 during continuous endotoxin infusion in comparison to bolus administration. IL-10 is the archetypal anti-inflammatory cytokine which inhibits the production of TNF-α and IL-1β. IL-6 exerts both pro- and anti-inflammatory effects, as it not only activates B- and T-lymphocytes and triggers the acute phase response and the coagulation cascade, but also can inhibit production of TNF-α^21^. Therefore, the distinct surge in IL-10 and IL-6 levels may be part of the negative feedback mechanism that controls TNF-α and CXCL8. Understanding the orchestration of the pro- and anti-inflammatory balance is of great interest, as a shift towards anti-inflammation is observed in patients with ongoing systemic inflammation^23^. This shift is presumed to prevent collateral damage of the pro-inflammatory response, but can also result in loss of immunocompetence and subsequently increase susceptibility to secondary infections. In sepsis patients, this phenomenon is termed sepsis-induced immunoparalysis^24^, although it is also observed in patients following trauma^25^ and cardiac resuscitation^26^ within hours after the insult. Therefore, the disproportionate production of anti-inflammatory cytokines during continuous infusion may mirror inflammation-induced immunoparalysis observed in patients.

Two independent, unsupervised clustering methods yielded similar groups of cytokines. Although these analyses uncover the relationships between cytokines, they do not provide a theoretical framework to explain these relationships and need to be interpreted with caution. Nevertheless, some notable relationships are observed. First, the primordial anti-inflammatory cytokine IL-10 has its own cluster, which explains 10% of the variance of the data Second, the aforementioned pro-inflammatory cytokine TNF-α and the chemokine CXCL8 are closely linked. The third group consists of IL-1β and IL-6, which are both potent pyrogens. The last group consists of MIP-1α and MIP-1β, both chemokines produced by macrophages, which attract and activate granulocytes and stimulate the production of other cytokines. Perhaps not surprisingly, component 3, with pyrogens IL-6 and IL-1β correlates significantly with the development of fever and symptoms. A direct correlation between the level of cytokines and clinical outcomes was previously unidentified^10^. It appears plausible that, using exploratory factor analysis, we have increased the power to identify meaningful correlations by quantifying the response of each individual on factors, which are groups of cytokines that act alike, instead of the cytokines by themselves.

Endotoxemia models have limitations. First, endotoxemia is an experimental model of inflammation, which only embodies certain aspects of the pathophysiology of sepsis, trauma, burns, and major surgery. However, as these conditions are heterogeneous by themselves, their pathogenesis is difficult to study in patients. Therefore, endotoxemia models should be envisioned as a complementary method to study the pathophysiology of systemic inflammation, in addition to *in vitro, ex vivo* and animal studies, and clinical studies in patients. Second, we did not identify relevant changes in mean arterial blood pressure in comparison to the placebo group. This is in contrast to the findings of a previous study in which a bolus administration of 4 ng/kg EC-5 endotoxin resulted in a profound hyperdynamic state with depressed left ventricular ejection fraction and reduced myocardial contractility^27^. Perhaps a bolus administration of 4 ng/kg, instead of a gradual infusion is required to produce such effects. The herein described models should therefore be used with apprehension to study hemodynamic changes in patients with systemic inflammation, and other models of sepsis may be more suitable for this purpose. We have not included a group in which a bolus injection of 4 ng/kg was administered to identify if differences between bolus and continuous infusion are the resultant of differences in dose or infusion rate. This limits the interpretation as to what extent the differences between the models are caused by dose or infusion mode.

The human model of systemic inflammation evoked by continuous infusion of endotoxin as described in the present study provides several new research opportunities. In bolus models, the inflammatory response comes and goes rapidly, limiting the possibility to demonstrate an effect of an intervention initiated following endotoxin administration. With continuous infusion, inflammation develops more gradually and is sustained for several hours, which extends the time window to demonstrate an effect of a therapeutic intervention, probably to a time point after initiation of endotoxin infusion. This is highly relevant, as in most patients with systemic inflammation, such as those suffering from sepsis, trauma or burns, there will be no opportunity for treatment before the inflammatory insult. Being able to demonstrate efficacy of an intervention administrated during inflammation would improve the clinical applicability. In addition, although still relatively short-lived, continuous endotoxin infusion may better represents the physiologic response of sustained immune triggering as observed in patients, including the emerging negative feedback mechanisms and development of endotoxin tolerance.

In conclusion, continuous infusion of endotoxin elicits a safe, reproducible, controlled, systemic inflammatory response in humans *in vivo,* which lasts for several hours. This model provides new options to study the innate immune response during continuous exposure to inflammatory stimuli. Furthermore, it offers a larger time window to evaluate immunomodulatory interventions following the onset of inflammation.

## Methods

### Study design

Data were obtained from the control arms of three randomized controlled studies, using placebo or endotoxin bolus administrations of 1 or 2 ng/kg, or a bolus administration of 1 ng/kg followed by continuous infusion of endotoxin at a dose of 1 ng/kg/h. Studies were performed after approval of the local ethics committee CMO Regio Arnhem-Nijmegen and are registered at ClinicalTrials.gov (NCT02642237 (December 2015); NCT02085590 (March 2014); NCT02612480 (November 2015), respectively). All study procedures were in accordance with the declaration of Helsinki, including the latest revisions.

The purpose of this report is to characterize a novel human endotoxemia model aimed at eliciting systemic inflammation in a more clinically relevant manner and potentially providing more opportunities for pharmacologic modulation. To provide an interpretational context, data of bolus injections of 1 and 2 ng/kg of the same endotoxin are also described. Because we did not include a group that received a bolus administration of 4 ng/kg (the same cumulative dose as used in the continuous infusion group), it needs to be stressed that differences between the continuous infusion group and the bolus groups may either originate from a difference in infusion rate or a difference in cumulative dose.

### Study protocols

Eligible subjects were healthy, non-smoking male subjects aged 18–35 years, with a normal physical examination, electrocardiography, and routine laboratory values at the screening visit. Exclusion criteria were pre-existent disease, febrile illness in the past four weeks and drug use. All subjects gave written informed consent to participate in the study. Before the experiment, subjects refrained from caffeine and alcohol for 24 h and from food and drinks for 12 h. Radial artery cannulation (Angiocath; Becton Dickinson, USA) facilitated blood pressure monitoring (Edward Lifesciences, Irvine, CA, USA) and blood withdrawal. A venous cannula was placed in an antebrachial vene for intravenous (i.v.) hydration and endotoxin administration. A three-lead electrocardiogram registered heart rate. All haemodynamic data was recorded with an interval of 30 seconds using an in-house developed system from a Philips M50 monitor (Eindhoven, The Netherlands).

To prevent vasovagal responses subjects were prehydrated by infusion of 1.5 L glucose 2.5%/NaCl 0.45% in one hour^28^. Purified lipopolysaccharide (LPS, US Standard *Escherichia coli* O:113 endotoxin) was obtained from the Pharmaceutical Development Section of the National Institutes of Health (Bethesda, MD, USA). The lyophilized powder was reconstituted in 5 mL NaCl 0.9% for injection and vortex mixed for 20 min. An i.v. bolus of endotoxin was administered at a dose of 1 ng/kg or 2 ng/kg at 0 h. Continuous infusion was initiated after a bolus of 1 ng/kg at 0 h, followed by a continuous infusion at 1 ng/kg/h for 3 h, resulting in a cumulative dose of 4 ng/kg shown to be safe in previous studies^10^,^11^,^12^. The placebo group received matched bolus volumes of vehicle (NaCl 0.9%) at 0 h. Hydration was continued with 150 ml/h for 6 h, and 75 ml/h during the rest of the experiment. Every 30 min, temperature was measured using a tympanic thermometer (FirstTemp Genius 2; Covidien, Dublin, Ireland) and flu-like symptoms (headache, nausea, shivering, muscle and back pain) were scored on a six-point scale (0 = no symptoms, 5 = worst ever experienced), resulting in a total symptom score range of 0–25.

### Cytokine analysis

Ethylenediaminetetraacetic-(EDTA) anticoagulated blood was collected at various time points, centrifuged (2000 g, 4 °C, 10 min) and plasma was stored at −80 °C until analysis. As plasma concentrations may differ between assay manufacturers and batches, we re-analyzed concentrations of TNF-α, IL-6, CXCL8, IL-10, IL-1β, MIP-1α, MIP-1β and MCP-1 in all samplesbatchwise in one run using a simultaneous Luminex assay (R&D systems; Abingdon Science Park, UK). The lower detection limits were as follows; TNFα 1,46 pg/mL, IL-1B 0,76 pg/mL, IL-6 1,76 pg/mL, CXCL8 1,41 pg/mL, IL-10 1.22 pg/mL, IFNy 0,66 pg/mL, MIP1a 74,4 pg/mL, MIP1B 34 pg/mL, IL-1RA 26 pg/mL and MCP-1 13,2 pg/mL. Cross-reactivity was below 0.5% for all the cytokines, and the interassay variation was below 17%.

### Leukocyte counts

Analysis of leukocyte counts were measured using routine methods also used for patient samples (flow cytometric analysis on a Sysmex XE-5000).

### Statistical analysis

Distribution of data was tested for normality using Shapiro-Wilk test. As all data were normally distributed, they are presented as mean ± SEM. Total cytokine production is expressed as the area under the time curve (AUC) and group differences were analyzed using unpaired Students t-tests. Differences between groups in kinetics were analyzed using two-way analysis of variance (ANOVA) (group x time). Within group comparisons were made using one-way ANOVA followed by Bonferroni post-hoc tests. Hierarchical clustering analysis (HCA) was used to analyze coherence between cytokines. HCA is an unsupervised method to represent the relationships among variables with the length of the branch reflecting the degree of similarity. AUCs of cytokines from the endotoxemia groups were log-transformed and normalized, after which HCA was performed using Euclidean distances and an average clustering algorithm (CIMminer, Genomics and Bioinformatics group, National Cancer Institute, Bethesda, MD, USA). This analysis identified clusters of the cytokines (AUCs) and subjects. Exploratory factor analysis (EFA) was performed to identify groups of cytokine responses that correlate to such an extent that they could be summarized into one new variable (a factor). Thereby EFA is an unsupervised method that identifies latent constructs (or factors) which can summarize data in factors that cannot be measured directly. These factors can be used to assess the coherence between cytokines and to assess the relation between clusters of cytokines with clinical parameters. EFA was performed on log-transformed cytokine AUCs using Oblimin rotation, as we assume that latent constructs of cytokine data may correlate and not be entirely independent. The scores of the subjects on the newly derived factors, which summarize all the cytokine data, were correlated with the AUC of symptoms, change in temperature, and change in heart rate using Pearson correlation analysis. Unless specified otherwise, statistical analyses were performed using Graphpad Prism version 5.0 (Graphpad Software, San Diego, CA, USA) and SPSS for Windows 22.0 (SPSS Inc, Chicago, IL, USA). P-values < 0.05 were considered statistically significant.

## Additional Information

**How to cite this article**: Kiers, D. *et al*. Characterization of a model of systemic inflammation in humans *in vivo* elicited by continuous infusion of endotoxin. *Sci. Rep.* **7**, 40149; doi: 10.1038/srep40149 (2017).

**Publisher's note:** Springer Nature remains neutral with regard to jurisdictional claims in published maps and institutional affiliations.

## Figures and Tables

**Table 1:** Demographic characteristics of subjects.

	placebo n = 15	bolus 1 ng/kg n = 10	bolus 2 ng/kg n = 15	bolus 1 ng/kg + continuous 3 ng/kg n = 10	p-value
Age [yrs]	21.5 ± 0.5	21.1 ± 0.9	21.7 ± 0.7	23.6 ± 1.6	0.30
Height [cm]	181 ± 2	185 ± 2	185 ± 2	182 ± 2	0.40
Weight [kg]	77 ± 3	78 ± 3	78 ± 3	77 ± 4	0.98
BMI [kg/m^2^]	23.4 ± 0.9	22.8 ± 0.7	23.0 ± 0.8	23.2 ± 0.7	0.96

Data were obtained during screening visit and are presented as mean ± SEM. P-values were calculated using one-way ANOVAs. yrs: years, cm: centimeter, kg: kilogram, BMI: body mass index, m: meter.

**Table 2:** Exploratory factor analysis pattern matrix.

	Factor 1	Factor 2	Factor 3	Factor 4
**% of variance explained**	64.5%	10.9%	9.2%	7.5%
**CXCL8**	0.999			
**TNF-α**	0.805			0.189
**MCP-1**	0.472	0.213	−0.255	0.256
**IL-10**		1.010		
**IL-1β**			−1.008	
**IL-6**	0.243	0.262	−0.688	
**MIP-1β**				0.989
**MIP-1β**	0.302			0.730

Pattern matrix after oblimin rotation with Kaiser Normalisation as obtained from EFA on log-transformed normalized area’s under time-concentration curves of cytokines after endotoxin administration. Only loadings >0.1 are displayed.
